# Optimizing Newborn Outcomes in Cesarean Sections: A Comparative Analysis of Stress Indicators under General and Spinal Anesthesia

**DOI:** 10.3390/children11070783

**Published:** 2024-06-27

**Authors:** Anna Uram-Benka, Izabella Fabri-Galambos, Marina Pandurov-Brlić, Goran Rakić, Nikola Bošković, Jasminka Uram-Dubovski, Jelena Antić, Dejan Dobrijević

**Affiliations:** 1 Institute for Children and Youth Health Care of Vojvodina, 21000 Novi Sad, Serbia; 2 Faculty of Medicine, University of Novi Sad, 21000 Novi Sad, Serbia; 3 Obstetrics and Gynecology Clinic, Clinical Center of Vojvodina, 21000 Novi Sad, Serbia

**Keywords:** newborns, cesarean section, anesthesia

## Abstract

Background and Objectives: The moment of birth represents a complex physiological process that is followed by adaptive changes in the vital systems of the newborn. Such reactions have their positive but also negative effects. The aim of this research was to determine the difference in laboratory values of stress indicators in newborn children delivered by cesarean section (CS) with general and spinal anesthesia. We attempted to make a recommendation about the optimal type of anesthesia based on our results. Materials and Methods: The study was performed on 150 healthy term newborns delivered by urgent or planned CS. Samples for adrenocorticotropic hormone (ACTH), cortisol, triglycerides, and interleukin-6 (IL-6) were analyzed. Results: Leukocyte numbers, triglycerides, and blood sugar values were normal for the newborns’ age, with statistically significantly lower values of blood sugar and triglycerides in newborns delivered by CS in spinal anesthesia (*p* < 0.005) compared to general anesthesia. There were no significant differences in ACTH, cortisol, and IL-6 levels between those newborns delivered via CS after spinal or general anesthesia. Conclusions: In cases where vaginal delivery is not possible, when CS is indicated, the use of well-controlled spinal anesthesia is followed by lower degrees of metabolic, inflammatory, and stress responses and better vitality of the baby upon birth.

## 1. Introduction

The moment of birth represents an important, profound, and transformative experience for mothers, which is the beginning of the new chapter in life. Simultaneously, for the newborn, it represents a remarkably challenging physiological process characterized by dynamic and complex changes in various systems, such as respiratory, cardio–circulatory, renal, and gastrointestinal, ultimately leading to the crucial adaptation of thermoregulation [1]. These physiological moments underscore the significance of childbirth as a crucial event in the life cycle, marking the short-term and long-term health of both the mother and the newborn.

The mode of delivery influences various metabolic and hormonal processes in the newborn, which trigger fluctuations in the levels of numerous proinflammatory, metabolic, and hormonal markers. Existing research reveals that these marker levels tend to peak during vaginal delivery, reflecting the intense physiological responses associated with this mode of birth. Conversely, levels are comparatively lower during planned elective cesarean sections (CS), indicating a distinct profile of responses in this controlled setting [2]. Epidural analgesia has been associated with alterations in fetal stress markers, highlighting its potential impact on neonatal outcomes [3]. This interplay of reactions has both positive and negative effects, which is why meticulous study and analysis are needed. Understanding these effects is very important for optimal decision-making in individual cases, as the choice of delivery mode significantly influences the short-term health and future well-being of both mother and newborn.

In the domain of newborn delivery, the roles of general anesthesia (GA) and regional anesthesia play a pivotal role in marking the birthing experience. GA is typically employed when a CS is mandated or in specific medical conditions or emergencies [4]. However, emerging studies are emphasizing the benefits of regional anesthesia, particularly neuraxial methods such as epidural or spinal anesthesia (SA). These methods represent ways to improve outcomes for both maternal and neonatal health. Neuraxial anesthesia is commonly used for pain management in the different stages of labor and delivery. However, this mode of anesthesia not only provides effective pain relief but also makes it possible for the mother to remain awake and actively participate in the birth process. This way, mothers are continuously involved in the process [5].

The advantages of regional anesthesia extend beyond maternal comfort and involvement and positive impact on the newborns’ outcomes. Some data indicate that regional anesthesia for CS deliveries is associated with reduced respiratory depression in the newborn. It is even superior to well-established APGAR scores compared to GA [6]. Additionally, regional anesthesia facilitates immediate skin-to-skin contact between the mother and newborn. This promotes early bonding and initiates breastfeeding, which are crucial elements for the health and well-being of the neonate [7].

A notable study by Iddrisu and Khan contributes to this discourse, deeming both anesthesia techniques reliable and well-tolerated during cesarean delivery. However, regional anesthesia emerged as the preferred option for elective CSs, beacause it has demonstrated superior benefits for maternal and fetal outcomes when compared to GA [8]. This insight highlights the importance of careful anesthesia choices for the specific needs of each delivery, ensuring the best possible outcomes.

This exploration into the dynamics of childbirth, its metabolic processes, and anesthesia types describes the multifaceted nature of obstetric care. As we strive for advancements in personalized medicine in general and so in obstetrics and obstetrics anesthesia, a comprehensive understanding of these factors becomes crucial. Through our ongoing research, we aim to contribute not only to the immediate practices in the delivery room but also to the broader conversation surrounding personalized care and its implications for the long-term health of both mothers and newborns [9].

Stress indicators in the newborn could be useful to analyze the different types of anesthesia during CS because they precisely and objectively measure the level of stress experienced during different types of anesthesia as well as the safety of newborns under various anesthesia methods. By assessing stress parameters, it becomes feasible to reliably determine which type of anesthesia is more favorable and safer for newborns [7,8,9].

The objective of this research was to determine the differences in laboratory parameters, specifically stress indicators, among neonates delivered via CS under either general or spinal anesthesia. Furthermore, the obtained results were compared with neonates undergoing vaginal delivery. This analysis aimed to create evidence-based recommendations pertaining to the optimal choice of anesthesia modality in the context of elective CS.

## 2. Materials and Methods

A study of a total of 150 infants of both genders was carried out. This is a prospective observational study, carried out at the Clinical Center of Vojvodina in Novi Sad, Serbia, including all consecutive women that went to the gynecology and obstetric clinic for delivery between the 1st of September and the 31st of December 2012.

### 2.1. Sample Selection

In total, 150 expectant mothers were enrolled in the research project between September and December 2012. All women willingly participated after receiving a thorough explanation of the study’s objectives and procedures. The participants were actively engaged in the decision-making process regarding the mode of delivery, as the study excluded emergency deliveries. Written consent was obtained from all participants, i.e., mothers, by the attending anesthesiologist.

The inclusion criteria in this study were as follows: the health of both the mother and the fetus, a gestational age between 37 and 41 weeks, and a birth weight from 2500 to 4000 g. The birth weight was estimated using methods such as ultrasound measurements, fundal height assessment, maternal weight gain monitoring, and clinical evaluation and confirmed after the delivery. Exclusion criteria for mothers included chronic illness, anemia, in vitro fertilization, twin pregnancies, preeclampsia, eclampsia, obesity, hypertension, abnormal glucose tolerance testing results, and inadequate prenatal care. Newborn-related exclusion criteria included fetal distress confirmed with cardiotocography, gestational age below 37 weeks, prenatally diagnosed anomalies, intrauterine growth restriction, emergency CS delivery, difficulty with transition, respiratory distress, and admission to the neonatal intensive care unit. Emergency CSs were not included in the study because they were indicated either due to maternal illness or fetal distress, which could impact stress indicators.

We included a total of 150 healthy newborns with gestational ages ranging from 37 to 41 weeks in the study. These participants were categorized into three groups based on the method of their mother’s delivery and the type of anesthesia administered—those delivered through elective CS under general anesthesia (GA group), those delivered via elective CS with spinal anesthesia (SA group), and those delivered vaginally (VD group). The determination of participants in each group was conducted using the normal approximation method, employing the Z-test statistic (n = 45). The first group comprised 50 newborns delivered through elective CS under general anesthesia (GA group), the second group consisted of 50 neonates delivered via elective CS with spinal anesthesia (SA group), while the third group comprised 50 neonates delivered through spontaneous vaginal delivery (VD group).

**Figure 1:**
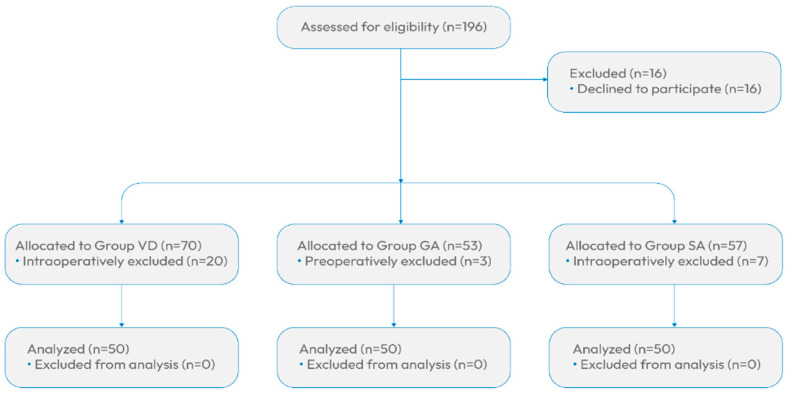
Participant inclusion criteria diagram.

For our research, we initially identified 196 women in labor. However, 16 pregnant women declined to participate, resulting in a total of 180 women who provided informed consent and were included in the study. These participants were divided into three groups based on their planned delivery method: vaginal delivery, delivery under general anesthesia, and delivery under spinal anesthesia. In the VD group, 20 pregnant women were excluded during the procedure. Twelve of these women had initially planned for a VD but required an emergency CS due to delivery complications. Additionally, eight pregnant women were excluded because their deliveries were not completed during the morning shift when data collection took place. In the GA group, three pregnant women withdrew from the study just before surgery. In the SA group, seven pregnant women were excluded intraoperatively because SA was insufficient, necessitating the application of analgosedation instead (Figure 1).

### 2.2. Anesthesia Administration

Spinal anesthesia (SA) was administered by injecting 3 mL of 0.5% bupivacaine, utilizing a “single-shot” technique. For patients undergoing general anesthesia, a regular premedication was administered, which included the following: 50 mg of ranitidine, metoclopramide (10 mg), and diazepam (5 mg). The induction phase of anesthesia included the IV administration of 1mg of midazolam, propofol (in the dose of 2.5 mg per kg), and suxamethonium (1 mg per kg). The maintenance phase was managed by the usage of the following combination: sevoflurane with a 0.5 minimum alveolar concentration (MAC) of 0.1, a gas mixture of nitrous oxide (N_2_O) and oxygen (O_2_) in equimolar proportions (1:1), and rocuronium bromide (in the dosage of 0.15 mg/kg). Airway management included endotracheal intubation for GA. It is very important to mention that all deliveries were conducted without any complications under elective anesthesia after the mothers’ agreement.

### 2.3. Laboratory Analyses

Blood samples for biochemical analysis were obtained from the umbilical artery right after umbilical cord clamping. There were no delays in the clamping process. EDTA–containing vacuated tubes (Becton Dickinson, San Antonio, TX, USA) were used for blood collection and to prevent blood clotting during the process. Right after, the blood samples were centrifugated (Hettich, ROTOFIX 32A, Burladingen, Germany) at 2400 rotations per min for 5 min for biochemical analyses.

The leukocyte count was determined by the Advia 2120i hematology analyzer (Siemens, Germany), which uses standard and well-established technique of flow cytometry after the specific staining.

For the determination of triglycerides, a standard biochemical photometry technique was employed. This was performed on the clinical chemistry analyzer AU 480 (Beckman Coulter, Nyon, Switzerland). The method used for determination of triglyceride levels is GPO-PAP, which stands for glycerol-3-phosphate oxidase (GPO) and para-amino-phenazone (PAP). In this method, GPO is acting on glycerol-3-phosphate, and in the process, hydrogen peroxide is produced. In the next reaction, enzyme peroxidase is acting on the hydrogen peroxide and color-producing substrate, which results in the change of the color of the solution. The intensity of the color can be measured photometrically and is directly proportional to the concentration of the analyte.

The analysis of adrenocorticotropic hormone (ACTH) and cortisol levels was conducted using the electrochemiluminescence immunoassay (ECLIA) method by a clinical immunochemistry analyzer Cobas e411 (Roche, Basel, Switzerland). It is a very common laboratory technique used in clinical diagnostics laboratories to measure the concentration of specific biomolecules in biological samples. This method combines elements of electrochemistry and luminescence to detect and quantify analytes such as hormones, proteins, or other substances of interest. In an ECLIA, the target biomolecule (e.g., a hormone like ACTH or cortisol) is bound to a specific antibody labeled with a substance that emits light when exposed to an electrical current. The emitted light is then measured, and its intensity correlates with the concentration of the biomolecule in the sample. This technique offers high sensitivity and a broad dynamic range, making it suitable for accurately measuring low concentrations of analytes in complex biological samples. ECLIA has become a widely used method in clinical laboratories due to its precision, reliability, and ability to provide quantitative results for a variety of biomarkers. In the context of this paper, the use of ECLIA for analyzing adrenocorticotropic hormone (ACTH) and cortisol levels underscores the commitment to employing advanced and precise methodologies for evaluating stress indicators in neonates delivered via different anesthesia modalities.

The enzyme-linked immunosorbent assay (ELISA) method was utilized for assessing interleukin-6 (IL-6) levels. In an ELISA for IL-6 determination, microtiter plates are coated with a specific IL-6 capture antibody. After blocking non-specific binding sites, test samples and IL-6 standards are added, allowing captured IL-6 to form a sandwich complex with a biotinylated detection antibody. Streptavidin-HRP is then introduced to enable an enzymatic reaction with a chromogenic substrate, resulting in a colored product. The color intensity, directly proportional to IL-6 concentration, is measured photometrically. Standard curves generated from known IL-6 concentrations aid in determining the cytokine levels in test samples, making ELISA a widely used and sensitive method for cytokine quantification in biological samples.

### 2.4. Statistical Analyses

The statistical analysis of the collected data was conducted with meticulous attention to detail, employing the widely recognized SPSS 23.0 package for Windows. In our pursuit of a thorough understanding, descriptive statistical methods were applied to compute a spectrum of parameters, including average values, standard errors, standard deviation, skewness, and kurtosis, as well as the standard errors of skewness and kurtosis.

For the investigation of variations in average values pertaining to parametric variables, Student’s *t*-test was employed, providing a robust assessment of significance. Non-parametric data, on the other hand, underwent scrutiny through the chi-squared test, allowing for a comprehensive analysis of categorical variables. Throughout these analyses, stringent significance levels were established at *p* = 0.05, *p* = 0.01, and *p* = 0.001, ensuring a meticulous evaluation of statistical significance.

In our pursuit of uncovering nuanced insights, the presence and significance of differences in observed average values among groups were explored through one-way factor analysis (ANOVA). This advanced statistical method allowed for a comprehensive examination of potential group variations. To further enhance our understanding, the LSD Post Hoc test was subsequently applied, enabling a nuanced assessment of specific group pairs and providing deeper insights into potential variations that may exist within the dataset.

To determine the sample size, a calculator incorporating various combinations of confidence, precision, and variability was employed. Cochran’s equation was utilized for the calculation process:

$$n_{0}=\frac{Z^{2}p(p-1)}{d^{2}}$$

where *Z* represents the *Z* statistic corresponding to the chosen confidence level, *p* stands for the anticipated prevalence or proportion, and *d* represents the desired precision level. When dealing with smaller populations of a known size, the sample size is determined by applying Cochran’s equation along with a population correction. This correction accounts for the size of the known population in the calculation process:

$$n=\frac{n_{0}}{1+\frac{\left(n_{0}-1\right)}{N}}$$

where *N* represents the population size, specifically the annual number of childbirths. The determined minimal sample size that appropriately considers the population size, along with the specified precision, confidence, and variability combination, is 136. However, we included a total of 150 healthy newborns with gestational ages ranging from 37 to 41 weeks in the study.

### 2.5. Ethical Approval

Approval for this study was granted by the Ethics Committee of the University Clinical Center of Vojvodina, Novi Sad, Serbia, under the reference number 00–08/466. Prior to participation, written informed consent was secured from all individuals involved, specifically the mothers participating in the study.

## 3. Results

Our study included 150 newborns, segmented into three distinct groups: GA, SA, and VD. Fundamental characteristics of newborns in each group are delineated in Table 1 for comparative evaluation.

**Figure 2:**
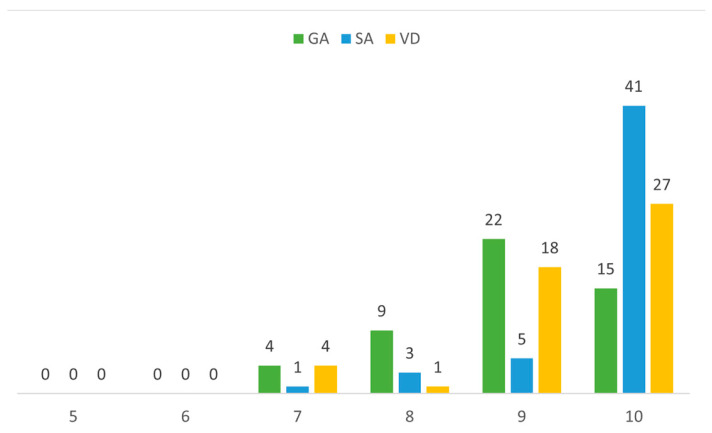
Apgar scores at 1 min in various delivery modes.

Apgar score assessments conducted within the first minute revealed significantly elevated values among neonates delivered via CS under spinal anesthesia in comparison to those born under general anesthesia (*p* < 0.001) and those delivered through spontaneous vaginal delivery (*p* = 0.005). Notably higher scores observed among neonates delivered via maternal spinal anesthesia compared to those delivered under general anesthesia (*p* < 0.001) (Figure 2).

### 3.1. Laboratory Analyses

The results of laboratory analyses encompassing leukocyte counts, glucose levels, triglycerides, cortisol, adrenocorticotropic hormone (ACTH), and interleukin-6 (IL-6) are shown in Table 2.

#### 3.1.1. Leukocytes

Leukocyte values exhibited a statistically significant elevation in subjects born through spontaneous vaginal delivery compared to the cohorts delivered through elective CSs, whether under general or spinal anesthesia (*p* < 0.001). This observation extended to monocyte counts (*p* < 0.05) and granulocyte counts (*p* < 0.001) as well. Notably, lower levels of both monocytes and granulocytes were discerned in neonates delivered under general anesthesia, although statistical significance was achieved solely in the case of monocytes (*p* < 0.05) when compared to those born under spinal anesthesia.

#### 3.1.2. Glucose

Blood glucose levels were observed to fall within the expected range for subjects of the corresponding age demographic. Notably, the highest blood glucose values were documented among neonates belonging to Group VD, whereas the lowest levels were observed in neonates within Group SA. Significantly elevated blood glucose levels were evident in neonates delivered under GA compared to those born under spinal anesthesia, demonstrating statistical significance (*p* < 0.01).

#### 3.1.3. Triglycerides

Systematic monitoring of triglyceride levels in newborns revealed notable disparities between the observed groups, indicating statistically significant differences (*p* < 0.001). Specifically, neonates delivered through spontaneous vaginal delivery exhibited significantly elevated triglyceride levels compared to those delivered via CS, irrespective of the type of anesthesia administered (*p* < 0.001). Furthermore, newborns delivered under general anesthesia demonstrated significantly higher triglyceride values (*p* < 0.05) than their counterparts delivered under spinal anesthesia.

#### 3.1.4. Cortisol

The cortisol levels of participants in Group VD exhibited a significant elevation (*p* < 0.001) compared to neonates delivered via CS under general and spinal anesthesia. Conversely, distinctions in cortisol concentrations between Groups GA and SA were found to be statistically nonsignificant (*p* > 0.05).

#### 3.1.5. ACTH

Group VD exhibited markedly elevated ACTH values in comparison to groups GA and SA, revealing a statistically significant difference (*p* < 0.001). However, no substantial distinctions were observed in ACTH levels between neonates delivered via CS under either spinal or general anesthesia (*p* > 0.05).

#### 3.1.6. IL-6

The mean IL-6 concentration in the VD group is significantly higher (*p* < 0.001) in comparison with the newborns delivered via CS (GA and SA). The disparity in concentrations between the GA and SA groups is statistically nonsignificant (*p* > 0.05).

## 4. Discussion

Childbirth represents a distinctly stressful physiological event for both the parturient and the neonate [10,11,12]. Prevailing in professional discourse and clinical practice is the perspective endorsing the execution of CS under regional anesthesia, predominantly SA [13]. Contemporary investigations highlight a potential neurotoxic impact of anesthetics on fetal and neonatal development, positing detrimental consequences for various facets of child development [14,15]. The cumulative neurotoxic effect of anesthetic agents is hypothesized to pose a risk to developing nerve structures, suggesting the desirability of minimizing the number of anesthetic drugs administered [16]. It is noteworthy that regional anesthesia circumvents the utilization of numerous potentially harmful drugs [17].

In the late stages of pregnancy and during parturition, there is a discernible elevation in blood glucose levels by the action of catecholamines and cortisol, principally through gluconeogenesis and glycogenolysis. Analysis of umbilical blood glucose levels revealed adherence to reference parameters relative to age, a noteworthy observation with profound implications for meeting the energetic demands of neonates. Particularly, a substantial elevation in these values was observed in neonates delivered through natural childbirth, indicative of a heightened modulation of hormonal factors instigating blood glucose elevation via gluconeogenesis and glycogenolysis in the context of CS deliveries with anesthesia. Neonates delivered via CS under GA exhibited significantly elevated blood glucose levels (*p* < 0.01) compared to those delivered under spinal anesthesia, although both findings remained within the spectrum of reference values. These findings align with a study by Kayiran and Gürakan involving 1540 term-delivered newborns, wherein neonates born vaginally manifested significantly higher blood glucose levels than those delivered via CS [18].

Perinatal stress induces a rise in maternal blood lipid levels, consequently impacting umbilical blood composition, attributed to the influence of catecholamines and glucagon, which augment lipase activity. The concurrent triglyceride levels in subjects born through natural delivery exhibited a statistically significant elevation compared to neonates delivered via CA, encompassing both GA and SA. No discernible differences in triglyceride levels were identified between neonates delivered under GA or SA [18,19]. Our findings underscore the significant impact of delivery mode on triglyceride levels, whereas the type of anesthesia employed for CS procedures did not impart discernible distinctions in triglyceride outcomes.

ACTH and cortisol exhibit interdependence through the hypothalamic–pituitary–adrenal axes, a pivotal physiological axis with significance both in the context of parturition and the preparation of the neonate for extra-uterine life. Antecedent to delivery, fetal blood levels of corticotropin-releasing hormone (CRH) and ACTH experience an elevation, paralleled by an increase in cortisol levels that culminate three days before delivery, bearing significance for the initiation of parturition [20,21]. Elevated cortisol values contribute to enhanced neonatal vitality postpartum. Cortisol levels are contingent upon the degree of perinatal stress, with a subordinate influence attributed to the mode of anesthesia employed during delivery [22]. Maximal cortisol concentrations are noted in prolonged VD, particularly those necessitating instrumental assistance [21]. In our study, natural childbirth is associated with significantly elevated ACTH and cortisol levels compared to the mean values observed in neonates delivered via CS, aligning with findings reported by other investigators [23]. Disparities in ACTH and cortisol concentrations between neonates delivered via CS under GA or SA were statistically nonsignificant, albeit lower cortisol values were discerned in the cohort subjected to SA. This observation aligns with prevailing literature, wherein maximal hormone values are reported in VD, particularly in challenging labor, and are comparatively diminished in elective CS [21]. Contrasting results have been reported by Ochedalski et al., who observed cortisol and CRH elevations in elective CS exceeding those in oxytocin-induced vaginal deliveries [24].

Leukocyte concentrations in the neonatal cohort under investigation remained within the stipulated reference parameters corresponding to the subjects’ respective age groups. Markedly elevated levels were observed in neonates delivered through natural childbirth compared to the two other delivery modalities [25]. The existing literature indicates the immunological significance of VD, wherein the stress inherent in the process induces structural and functional alterations in fetal leukocytes, affording protective mechanisms against infections. The augmented propensity for atopic diseases observed in children delivered via CS may be attributed to these immunological considerations [26]. Notably, neonates delivered under GA exhibited reduced numbers of leukocytes in comparison to those delivered under SA. Elevated leukocyte values may also serve as indicators of intrauterine infections, such as chorioamnionitis. In our study, the absence of clinical signs of infection during the neonates’ maternity ward stay precludes the influence of prenatal and perinatal infections on leukocyte and IL-6 values [27,28].

IL-6 stands as one of the most potent cytokines during pregnancy, exerting considerable influence on cytotrophoblast proliferation. Placental cells are recognized producers of IL-6 and other pro-inflammatory cytokines; however, the precise mechanisms governing their transplacental passage remain elusive. Maternal illnesses, duration and mode of delivery, anesthesia type, and labor-induced stress collectively impact interleukin levels [29]. Some authors posit an association between prolonged labor and heightened IL-6 levels in both maternal and neonatal compartments [30]. IL-6 and C-reactive protein (CRP) levels serve as indicators of stress during delivery, yet discerning the specific stressor, be it premature birth, prenatal maternal infections, or intrapartum infections, remains challenging [31]. The presence of IL-6 in umbilical blood is normative during uneventful term deliveries, with elevated values indicative of potential infection. In response to fetal stress, the placenta and fetal membranes may release IL-6 as part of the inflammatory cascade, leading to elevated levels in umbilical blood. Besides potential infection, the mechanical stretching of the cervix and uterus during labor can trigger an inflammatory response, leading to the release of cytokines such as IL-6 [32]. In our study, neonates born through natural delivery exhibited significantly elevated IL-6 levels compared to the other cohorts, aligning with the established literature [31,32]. While IL-6 values in neonates delivered via CS under SA were lower than those under GA, the difference did not attain statistical significance. Comparable findings have been reported in studies examining IL-6 levels in maternal blood during delivery [33,34,35].

Neuroaxial anesthesia, a method administered while the parturient is conscious, facilitates early maternal–newborn bonding [36] and concurrently mitigates the neonatal exposure to potential neurotoxic effects associated with medications utilized in GA. The reduction in maternal morbidity and mortality is attributed to a diminished incidence of complications such as aspiration, failed intubation, inadequate ventilation, and subsequent hypoxic damage. These collective advantages contribute to the safeguarding of neonates during the delivery process [14]. Presently, the precise impact of drugs administered during GA on the neonatal brain and subsequent cognitive function remains insufficiently elucidated, underscoring the rationale for minimizing their usage whenever feasible. Neuroaxial anesthesia serves as an efficacious means to achieve this objective. The observed advantages of neuroaxial anesthesia in facilitating early maternal–newborn contact, reducing neurotoxic medication exposure, and decreasing maternal morbidity underscore its clinical significance [36].

In our study, SA was administered without adjuvants, a decision made to minimize potential confounders and standardize the anesthesia procedure across our study cohort. It is important to mention that adjuvants in SA could potentially prolong the anesthesia’s beneficial effects and mitigate complications such as maternal hypotension, potentially leading to positive influences on neonatal outcomes [37,38].

However, the values of the analyzed parameters are influenced by a multitude of factors beyond the scope of our current study. For instance, Hashiguchi et al. [39] explored the utility of heart rate variability (HRV) as a stress index in neonates, demonstrating significant correlations between HRV parameters and salivary cortisol levels, thereby highlighting HRV’s potential as a non-invasive indicator of physiological stress in clinical settings. Additionally, research by Rakić et al. [40] investigated the impact of delivery type and obstetric anesthesia on oxidative stress levels in newborns, revealing distinct metabolic responses among neonates delivered vaginally, via CS under GA, and via CS under SA. These findings underscore the complex nature of neonatal stress responses influenced by various delivery and anesthesia modalities.

Our study has some limitations. First, it was conducted as a single-center, observational study. Second, the relatively small sample size employed in our study could potentially impact the applicability of our results.

## 5. Conclusions

Our study validates previous results regarding the several pivotal aspects related to the impact of delivery modalities and anesthetic approaches on neonatal outcomes. Notably, SA demonstrates a potential protective role, as evidenced by lower cortisol values and reduced leukocyte levels compared to GA during CS. However, while lower leukocyte levels may suggest a reduced inflammatory response, excessively low levels could potentially indicate immunosuppression or increased susceptibility to infection. Therefore, the conclusion about the protective role of SA should be interpreted cautiously and may warrant further investigation in future studies to better understand its implications for neonatal outcomes. SA avoids the depressant effects of GA and opioids on the fetus, thereby improving adaptation to extrauterine life. A better Apgar score at birth after SA indicates the depressive effects of GA.

Based on this, the safety of both the mother and the newborn is enhanced with spinal anesthesia, demonstrating its protective role. Our study highlights how different delivery approaches and anesthesia affect newborns. Natural childbirth causes higher stress and immune responses. Spinal anesthesia seems to protect against this, showing lower stress levels during cesarean sections compared to general anesthesia. Neuroaxial anesthesia has benefits like promoting early bonding and reducing complications. These findings help improve delivery practices for both mothers and newborns. Further research is needed to understand long-term effects on newborn development.

## Figures and Tables

**Table 1:** General data about the newborns as divided in groups.

Parameter	VD	GA	SA	*p*-Value
Gestation age (weeks)	39.71 ± 0.82	38.97 ± 0.51	39 ± 0.91	<0.05 *
Body weight at birth (g)	3450.60 ± 363.66	3507.40 ± 436.01	3404.54 ± 403.48	ns
Male gender	24 (48%)	26 (52%)	27 (54%)	ns
Female gender	26 (52%)	24 (48%)	23 (46%)	ns

Values are mean ± standard deviation and numbers (%). * statistically significant differences between VD and GA and VD and SA. ns—non-significant.

**Table 2:** Values for laboratory analyses of the newborns as divided in groups.

Parameter	VD Group		GA Group		SA Group	
	Average	95% CI	Average	95% CI	Average	95% CI
Leukocytes (10^9^/L)	14.6	13.46–15.74	11.81	10.67–12.95	12.14	11–13.28
Glucose (mmol/L)	4.44	3.3–5.58	3.74	2.6–4.88	3.09	1.95–4.23
Triglycerides (mmol/L)	0.34	0.31–0.37	0.27	0.24–0.3	0.23	0.2–0.26
ACTH (pmol/L)	151.17	145.48–156.86	93.89	88.2–99.58	89.42	83.73–95.11
Cortisol (nmol/L)	434.87	420.66–449.08	188.39	174.18–202.6	183.54	169.33–197.75
IL-6 (pg/mL)	0.68	0.65–0.71	0.14	0.11–0.17	0.11	0.08–0.14

## Data Availability

Data are available upon reasonable request.
